# Argument Structure and Morphological Factors in Noun and Verb Processing: An fMRI Study

**DOI:** 10.1371/journal.pone.0045091

**Published:** 2012-09-18

**Authors:** Gabriele Garbin, Simona Collina, Patrizia Tabossi

**Affiliations:** 1 Department of Psychology, University of Trieste, Trieste, Italy; 2 Department of Psychology, Suor Orsola Benincasa University, Naples, Italy; University of Cambridge, United Kingdom

## Abstract

In a functional MRI (fMRI) study, we have investigated the grammatical categories of object noun, event noun and verb in order to assess the cortical regions of activation supporting their processing. Twelve Italian healthy participants performed a lexical decision task. They had to decide whether a string was an Italian word or not. Words could be objects like *medaglia* (medal), or events like the noun *pianto* (cry); or the verb *dormire* (to sleep). Noun and verb comparison shows differences in regions of activation in the left Inferior Frontal cortex and in the extent of the same areas. We have found specific areas of activation for object noun, and similarities in the pattern of activation for event noun and verb. The activations induced by pseudowords highly resembled the areas activated by the corresponding word category. The implications of the results are discussed in light of the recent debate on the role of grammatical category in the brain.

## Introduction

Noun and verb are indicated as distinct grammatical classes across different languages.

In neuropsychology it has long been known that the mental processing of noun and verb involves different steps. This fact is immediately evident when language impairments are taken into account. For example, agrammatic patients often experience greater difficulties in the production of verbs rather than nouns, whereas anomic patients often show the opposite pattern [1]–[5]. On the basis of these results, grammatical classes have been considered as an organizational principle in the mental lexicon. A rich body of data suggests that verb-processing deficits are caused by damage in the left frontal/prefrontal and parietal cortex [6]–[11], whereas damage in the left temporal areas is generally responsible for noun deficits [7], [12]. However, the data are not completely consistent. For example, damage extending into the frontal cortex does not always result in verb impairment [13].

Neuroimaging studies are only partially consistent with neuropsychological observations. Verb processing is often associated, across studies, with the activation of frontal/prefrontal and parietal areas [14]–[17]; on the contrary, the search for specific cortical activation linked to noun processing does not always give a positive result. For example, Perani et al. [17] conducted a PET experiment using a lexical decision task and, while finding the expected activations for verb, they did not obtain any significantly activated areas for noun. In another PET study, Tyler et al. [18] did not observe significant activations for noun with a lexical decision task or a semantic category task. It should be added that they did not find specific areas of activation for verb either. Using a grammatical-class switching task, Berlingeri et al. [19] did not find specific areas of activation for noun.

Activation for noun has been shown by Fujimaki et al. [14] in a lexical decision task, by Shapiro et al. [12] in a word inflection task, by Warburton et al. [15] in a semantic fluency task and by Bedny & Thompson-Schill [20] in a semantic similarity judgment task. These studies confirm that specific cortical regions for noun processing can be identified, but their results do not agree on a same area.

The grammatical class of noun can semantically refer to at least two different categories: object noun and event noun. The latter refers to events, and its morphology may or may not recall the semantically related verb, like *the chase/to chase*, *the bombardment/to bombard, the theft/to steal*. In Italian, with very few exceptions, event nouns are nominalizations derived from verbs through suffixes (e.g. -*mento* in *bombardamento/*bombardment from *bombardare/*to bombard; -*sione* in *uccisione/*killing from *uccidere/*to kill), and therefore these nouns are, in general, morphologically complex.

According to most linguists, event nouns have the same semantic and thematic structure as the verbs from which they derive [21]. It is conceivable, therefore, that although they belong to the grammatical class of noun, their processing may recruit cortical areas that are also involved in the processing of verb. There is evidence in this direction. In a picture-word interference study conducted in Italian, Vigliocco, Vinson and Siri [22] asked their volunteers to name target pictures of events (e.g. riding) with an infinitive verb (e.g. *cavalcare/*to ride). Along with the target, participants were presented with a distracter word, which was either an event noun or a verb, and could be semantically close or far from the target (e.g. close noun: *nuotata/*swim; far noun: *cinguettio/*tweet; close verb: *andare/*to go; far verb: *ruggire*/to roar). The semantically close words produced a reliable interference effect on picture naming, regardless of grammatical class.

Neuropsychological evidence also suggests that patients may treat event noun and object noun differently. Collina et al. [2] investigated the performance of three Italian agrammatic patients with a selective verb deficit assessed with a test based on nouns always referring to objects (BADA, *Batteria per l’Analisi dei Deficit Afasici/Battery for the Analysis of Aphasic Deficits* [23]). Patients were asked to perform a picture naming task using either a noun or a verb. There were four sets of pictures, depending upon the words to be used to name them: object nouns (e.g. *medaglia/*medal), event nouns (e.g. *pianto/*crying, *inseguimento/*chase), one-place argument verbs (e.g. *dormire/*to sleep), and two-place argument verbs (e.g. *distruggere/*to destroy). As expected, errors in the production of object nouns were statistically fewer than in the production of verbs. In addition, patients made fewer errors with one-place than with two-place argument verbs. Interestingly, their performance was significantly better with object nouns than event nouns. The latter did not differ from verbs.

In a similar vein, Tabossi et al. [10] investigated the performance of an agrammatic patient (named CM) with selective verb impairment. In a series of comprehension and production tasks, CM performed better with object nouns than with either verbs or event nouns, and the difference persisted even when the morphological complexity of event nouns was taken into account.

In a PET study conducted in Italian [24], participants were asked to listen to blocks of words that were either verbs (e.g. *galoppa/*s/he gallops) or event nouns (e.g. *corsa/*run) and referring either to sensation (e.g. *solletico/*tickle) or motion (e.g. *giravolta/*twirl). The results indicated specific areas of activation for sensory words in the anterior temporal cortex and for motion words in the primary motor cortex. However, no significant difference was found specifically for noun and verb.

Likewise, in an fMRI study [25], participants had to name pictures of events using either a verb in the infinitive form (e.g. *mangiare/*to eat) or in the present third person singular (e.g. *mangia-*s/he eats), or using a noun (e.g. *mangiata/*eating). This task produced a global activation in the area of the left inferior frontal gyrus, but no specific areas of activation for noun and verb.

Both [24] and [25] agree that, once semantics has been taken into account, there is no evidence that there are two distinct networks in the brain for the processing of noun and verb.

Shapiro and colleagues proposed an alternative view [26], [12]. In a PET study [26], German-speaking participants were asked to produce either singular/plural nouns or first person singular/plural verbs. Nouns showed greater activation in temporal regions bilaterally, whereas verbs showed greater activation in frontal areas. These results were confirmed in a subsequent fMRI study. Across three experiments, English-speaking participants were asked to produce short phrases (e.g. *many doors, he sweeps*) in response to words (noun or verb) or pseudowords (to be used as noun or verb in the context). In comparison to verb, noun production elicited greater activation in the left inferior temporal lobe. In comparison to noun, verb processing produced greater activation in pre-frontal areas and in the left superior parietal lobule. This pattern was consistent for words and pseudowords, abstract and concrete words, and regularly and irregularly inflected words. The authors suggest that these areas are involved in the representation of core conceptual properties of noun and verb. These abstract properties may show that all nouns refer to an identifiable thing, whereas all verbs refer an event taking place in time. Although in their studies Shapiro et al. did not use event nouns, they explicitly claim that their abstract characterization of noun concepts includes abstract nouns, mass nouns, and nominalizations.

Thus, while according to Vigliocco et al. [24] nominalization should not give rise to specific areas of activation when compared to verb, according to Shapiro et al. [26] nominalization activates the same areas as object noun.

As already observed, nouns could also refer to events, and event nouns have not been systematically investigated, with few exceptions [19], [27], [28]. To investigate this issue, we have conducted an fMRI experiment in which Italian speakers were presented with three separate blocks of words and pseudowords: object nouns (e.g. *nuvola/*cloud) and pseudowords selected to resemble simple object nouns (e.g. *grincipe*), event nouns (e.g. *liberazione/*freeing) and pseudowords with event nouns suffixes (e.g. *sbinamento*), and verbs (e.g. *brillare/*to shine) and pseudoverbs with verb suffixes *-are*, *-ere*, *-ire* (e.g. *adevare*). Nouns and pseudonouns were in the singular form, and verbs and pseudoverbs were in the infinitive form. The three groups of words were matched for frequency, concreteness, imageability, familiarity, and syllable length. All pseudowords were legal.

The task was a lexical decision. There is debate about the sensitivity of the lexical decision task to study grammatical categories [29]. However, in Perani et al. [17], specific neural networks resulted activated by verb, which should not have been the case, if lexical decision did not recruit grammatical class information. Moreover, using this task, Fujimaki et al [14] found that noun compared to baseline mainly activated Broca’s area and the insula bilaterally, though more extensively in the left hemisphere, left posterior temporal area (both superior and inferior), left occipito/temporal sulcus and left precentral sulcus. On the other hand, activations for noun are not revealed with tasks clearly involving the retrieval of class information, such as the grammatical class switching task [19]. In the present study, the use of a lexical decision task shows some advantages: it does not explicitly involve the retrieval of class information; the depth of processing is ensured without the need of a syntactic cue, given the use of suffixation in Italian; the use of comparable suffixed pseudowords, combined with the blocked presentation of the different word types, may cue participants to the relevant dimensions.

If Vigliocco and colleagues’ hypothesis is correct, then no different areas of activation should be observed for event noun and verb. On the other hand, if Shapiro’s hypothesis is correct, noun classes should produce similar patterns of activation, and this activation should differ from that produced by verb.

## Results

### Behavioral Performance

Behavioral data on the correct execution of the task reveal that all subjects performed the three experimental conditions with a low number of errors, ranging from 0% to 5% across subjects (mean 2.3%). The errors for each experimental condition were 4% (N0), 1.5% (N1) and 1.5% (V). More errors were observed in pseudoword than in word presentation, with a rate of 8.25 (33 pseudowords mistaken for words, and 4 vice versa); the maximum number of wrong answers per stimulus among subjects was 2.

### fMRI Results

The results are reported in terms of anatomical label, activation center coordinates (x, y, z) in MNI space, Z-score, and corresponding Brodmann Area (BA) in the left (L) or right (R) hemisphere. Due to the relevant number of areas of activation, a full description of all the results is available as Supporting Information S1.

### Global Effect

The global effect for all classes of words, masked by the effect of pseudowords, includes activations in the left inferior frontal gyrus (BA 44L), bilateral superior temporal gyrus (BA 22), bilateral inferior parietal lobule (BA 40), and postcentral gyrus (BA 43).

### Activations Associated with Separate Classes of Words Masked by Pseudowords

Object nouns only activated the left inferior parietal lobule toward the postcentral gyrus (BA 40L).

Event nouns activated Brodmann areas 46/10 and 45 in the left IFG, the transverse temporal gyrus toward the postcentral gyrus bilaterally (BA 40/41/42), and the right middle temporal gyrus (BA 39R).

**Figure 1:**
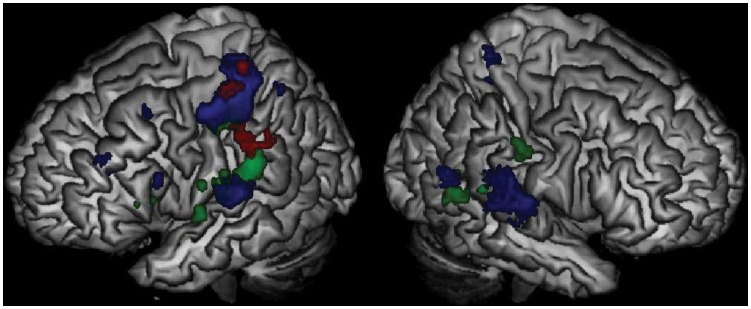
Separate activations for words masked by pseudowords. The results are superimposed on the same background. Color code: red: object noun (left IPL, −60 −32 32; Z = 3.10; BA 40L); blue: event noun (left IFG, −28 32 22; Z = 3.00; BA 46/10; −44 14 16; Z = 3.08; BA 45; bilateral transverse temporal gyrus, −62 −20 12; Z = 4.13; BA 40/41/42L; 60 −28 16; Z = 3.78; BA 40/41/42R; right middle temporal gyrus, 56 −58 14; Z = 3.85; BA 39R); green: verb (left IFG, −42 18 6; Z = 2.97; BA 44L/45L/13L; left IPL, −48 −32 22; Z = 4.16; BA 40L; left postcentral gyrus, −56 −6 16; Z = 2.99; BA 43L; bilateral superior temporal gyrus, −58 −4 4; Z = 3.24; BA 22L. 66 −52 10; Z = 3.21; BA 22R; right middle temporal gyrus, 40 −54 6; Z = 3.28; BA 37).

Verbs activated the left IFG (BA 44L/45L/13L), the left inferior parietal lobule (BA 40L) and postcentral gyrus (BA 43L), the superior temporal gyrus bilaterally (BA 22), right middle temporal gyrus (BA 37R). The activations resulting from the three conditions are superimposed on the same background in Figure 1.

### Comparison Among Classes of Words

The cross-comparison among the three classes revealed a higher number of activations. During analysis, we observed that the pattern of activation of pseudowords versus null baseline for each class partially recalled the pattern obtained for the corresponding class of words under the same condition. Cross-comparison therefore allowed us to identify areas which were previously shadowed by imposing mask and threshold on the map of activation of each class.

### Noun and Verb Comparison

**Figure 2:**
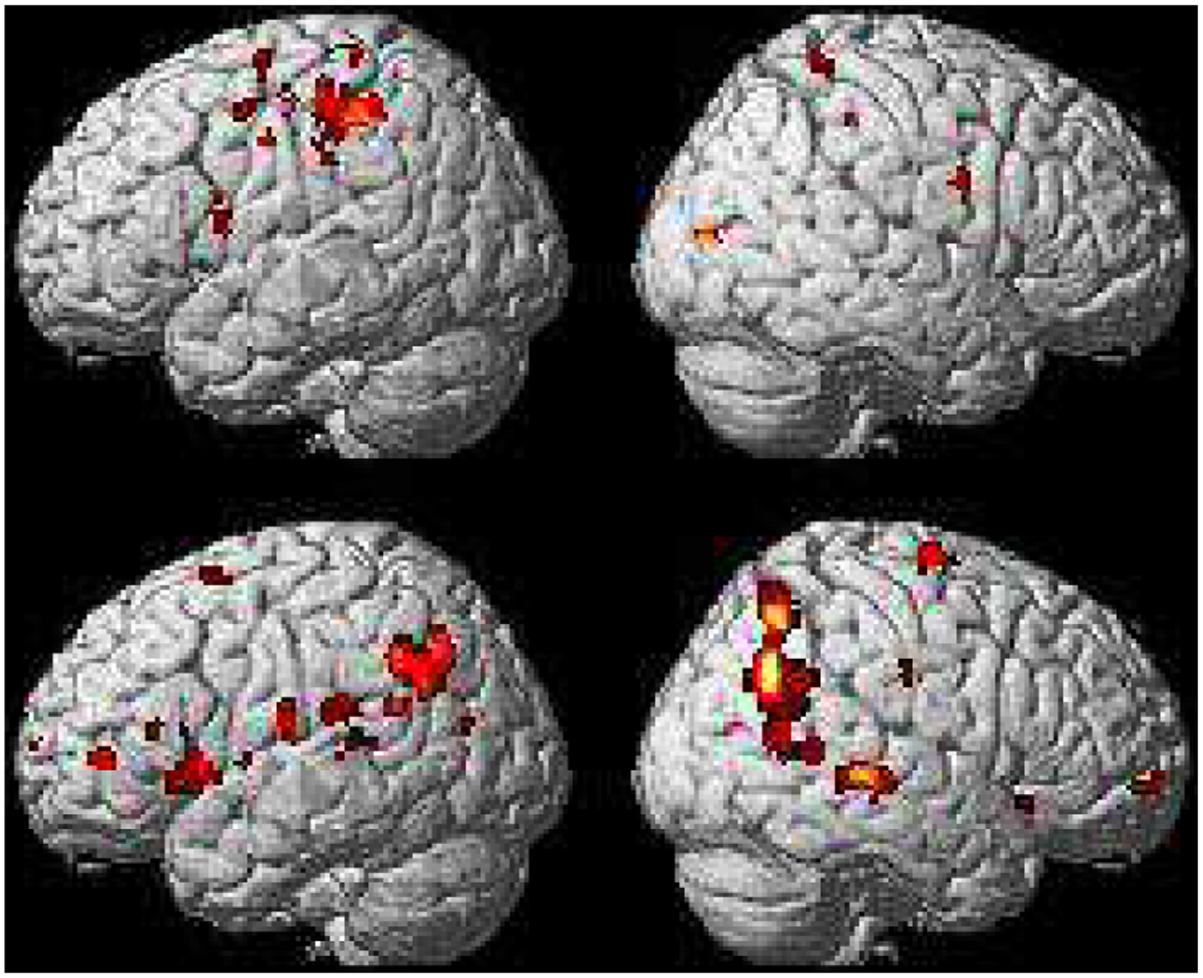
Object and event nouns masked by verb and vice versa. Nouns masked by verb show activations in the left insula (−34 −8 6; Z = 4.59; BA 13L), left IFG (−48 4 14; Z = 3.98; BA 44L), left inferior parietal lobule (−42 −32 46; Z = 4.92; BA 40L), left anterior cingulate cortex (−20 18 32; Z = 3.87; BA 32L), and right middle temporal gyrus (40 −82 8; Z = 5.07; BA 39R). Verb masked by nouns shows activations of interest in the left hemisphere (−44 18 0; Z = 4.18; BA 13L), left IFG (−32 26 −6; Z = 3.45; BA 47), (−42 42 6; Z = 4.25; BA 46), (−48 26 14; Z = 3.58; BA 46), (−8 66 8; Z = 3.71; BA 10), left ACC (−20 48 8; Z = 4.41; BA 32L), the left IPL and superior temporal gyrus (−48 −52 22; Z = 3.92; BA 40L. −66 −42 10; Z = 4.68; BA42L. −58 −16 18; 3.99; BA 43L. −60 −2 6; Z = 3.24; BA 22L), and the right hemisphere (38 20 −10; Z = 3.51; BA 47R. 28 56 −4; Z = 3.36; BA 10R. 64 −28 −2; Z = 5.07; BA 22R).

Compared to verb, noun elicits greater activation in a portion of the left insula (BA 13L) and IFG (BA 44L), the left inferior parietal lobule (BA 40L) and anterior cingulate cortex (BA 32L), plus the right middle temporal gyrus (BA 39R). In the opposite comparison, verb activates a wider portion of the left insula (BA 13L) and a number of areas in the left IFG including Brodmann areas 47, 46, 10, a portion of the anterior cingulate cortex (BA 32L) with a different center of activation from the previous comparison, the left inferior parietal lobule and superior temporal gyrus (BA 40L, 42L, 43L, 22L); the pattern on the right hemisphere is very similar (BA 47R, 10R, 22R) with the exception of BA 46 and 32. The results are shown in Figure 2.

### Noun Comparison

Object and event nouns share activations in same areas in the left IFG and parietal lobules, but with different centers and extent. Object noun activates Brodmann area 44 in the left inferior frontal gyrus, the insula (BA 13L), and the inferior parietal lobule bilaterally (BA 40). Event noun shows a similar pattern of activation (BA 44L, 13L, 40L, 40R), plus different activations in the left middle frontal gyrus (BA 46/10R), the right IFG (BA 44/45/46R, 13R), the superior temporal gyrus bilaterally (BA 41, 42, 22) and the left parahippocampal gyrus (BA 27/30L). The results are described in table 1.

**Table 1:** Activations of object noun versus event noun, and versus verb.

Object noun		Label	Area	x, y, z	K	Z
vs Eventnoun	L	Precentral gyrus	L44	−46 2 8	13	3.66
		Insula	L13	−36 −6 14	41	3.93
		Inferior parietal lobule	L40	−46 −60 46	469	5.64
	R	Inferior parietal lobule	R40	68 −32 34	24	4.59
		Cingulate gyrus	R24	4 −6 42	29	3.86
vs Verb	L	Insula	L13	−36 −8 16	31	3.77
		Precentral/inferiorfrontal gyrus	L44	−46 2 8	15	3.66
		Middle frontal gyrus	L6	−34 −4 52	258	4.90
		Inferior parietal lobule	L40	−42 −32 48	381	4.73
		Superior parietal lobule	L7	−16 −56 66	16	3.77
	R	Precentral/inferiorfrontal gyrus	R9/6	48 0 34	30	3.98
		Medial frontal gyrus/cingulate gyrus	R6/24	8 −12 54	158	4.61
		Inferior parietal lobule	R40	68 −32 34	27	4.59

### Object Noun and Verb Comparison

The patterns of activation of the two classes are almost entirely different; therefore the comparison reveals patterns recalling the results of the analyses of the separate classes. Masked with the map of activation for verb, object noun reveal an exclusive activation of Brodmann area 44L, which cannot be observed in the opposite comparison, and the insula. Conversely, verb elicits greater activation in areas 47 and 10 in the IFG bilaterally (BA 47, 10), left area 46, 45/13/47, the insula, the anterior cingulate cortex (BA 32/10L, 24L), and bilaterally the inferior parietal lobule (BA 39/40) and superior temporal gyrus (BA 22L, 39R).

### Event Noun and Verb Comparison

Compared to verb, event noun shows cortical activation in Brodmann area 44, 46 and the insula bilaterally, plus right area 45, the superior temporal gyrus bilaterally (BA 42) though more extensively in the right hemisphere, and the left parahippocampal gyrus (BA 27/30L). The opposite comparison results in the activation of the left IFG (BA 47L, 45L/13L, 10L), toward the anterior cingulate gyrus (BA 32L, 24L), and of the middle and superior temporal gyrus bilaterally (BA 22/42L, 22R, 39R).

### Comparison Between Object Noun and Event Words

The specific activation for object noun involves the inferior parietal lobule, postcentral gyrus and medial frontal gyrus bilaterally (BA40, 6), the insula (BA 13L), and the right anterior cingulate cortex (BA 24R). Event words, either verb or noun, recruit numerous areas bilaterally in the inferior frontal gyrus (BA 45, 46, 9, 13, 44), the anterior cingulate cortex (BA 32) and a relevant portion of the temporal lobe spreading towards the surrounding structures (BA 22, 40, 42).

## Discussion

In this study, we aimed to investigate the issue of the representation of grammatical classes in the mental lexicon. To this end, we performed a direct comparison involving object noun, event noun and verb.

The results of our study have evidenced specific areas of activation for object noun compared to verb, involving the inferior frontal gyrus, the left insula, and the left inferior parietal lobule. This pattern of activation contradicts the hypothesis according to which noun activation, when compared to verb, depends on the amount of morphological and syntactic processing required by the task [19]. In this study, the task did not require specific morphological and/or syntactic operations. We therefore hypothesize that the effect we observed reflects a genuine difference among the different classes of words.

Indeed, cross-linguistic variables could explain the magnitude of the differences in the observed patterns of activation. In English, grammatical class differences mainly emerge when morpho-syntactic operations are engaged. When a word is presented in isolation (e.g. walk) it can either be a noun or a verb. In order to solve the ambiguity, a morphological or syntactic context is necessary. In two lexical decision experiments, Vigliocco and colleagues found a grammatical class effect only when the primes, either verb or noun, were preceded by a grammatical context (a, to). However, when bare primes were presented, no grammatical class effect was observed. In a rich morphological language as Italian, this problem is strongly reduced and principally limited to verbs in the present form, third singular person, which may be morphologically identical to a noun (*porta*-s/he brings; *porta*-door).

We also found differences in the activation between object and event noun, suggesting that the class of nouns is not homogeneous. Moreover, event noun partially shares the same areas of activation involved in verb processing.

Event noun has many properties inherited from verb: semantically it refers to events and assigns thematic roles, and syntactically it selects complements and has an argument structure specifying the number of complements necessary for a well-formed sentence. However, from a grammatical point of view it is a noun. As its activations partially overlap those for verb, we suspect that a grammatical class distinction based on a simple noun/verb dichotomy could not be applied to the results of our study. In this respect, it is also difficult to explain the results on a semantic basis only. In the lexical decision task we used pseudowords with suffixes morphologically resembling the structure of Italian words. The activations found for pseudowords mostly overlapped those obtained for the homologous object nouns, argumental nouns and verbs. This pattern has strong analogies with the behavioral results observed by Shapiro and Caramazza [30]: the authors used a morphological transformation task in which, for example, a noun had to be transformed from singular to plural and a verb from present to past tense given a sentential context. Patient RC was impaired at inflecting verbs and pseudoverbs. On the contrary, patient JR was impaired at inflecting nouns and pseudonouns [13]. In addition, the results are in line with what observed by Shapiro [26]. Specifically, activation for verb and pseudoverb was found in the left rostral prefrontal cortex (in the left superior frontal gyrus), and for noun and pseudonoun in areas within the left and right temporal lobes. These areas have been reported to be, in turn, responsible for verb and noun processing. As we obtained a pattern of results similar to real words activations and in line with clinical observations, we should assume that our results cannot only be explained on the basis of a semantic distinction between objects and events. However, some authors argued that pseudowords should elicit some kind of meaning [31]. This objection mainly refers to a task including a sentential context. In our task, words were presented in isolation, thus avoiding any phrasal context that could elicit a meaning. Nevertheless, although a phrasal context was not given to the participants, the suffixes used to build the materials were plausible in Italian, especially in the case of argumental nouns and verbs. Therefore, it is possible that morphological factors may recall a word even when a pseudoword is processed and that this may reflect the pattern we observed. In addition, it could be argued that the pattern reflects a response based only on the stem itself, given that the affix is uninformative about the word status of the target. However, the pattern was observed not only for morphologically complex pseudowords (pseudoeventive nouns and verbs) but also for object nouns vs pseudonouns, in which the processing and morphologically biased response are less affected by morphological factors.

A possible explanation of our results involves properties shared by event noun and verb, namely syntactic and morphological factors. The event nouns used in the study are morphologically derived by verbs. It is possible that the partial overlap we observed in the activation areas are also due to the complex morphological processing of these words, which is, in terms of complexity, more similar to that of verbs. The same may happen for the structural properties of these words. Event noun inherits from the original verb a thematic grid and a subcategorization frame, which determine, in turn, any thematic roles and complements acquired. These factors may contribute to the activation of certain similar circuits when event noun and verb are considered. The results we observed are not against the idea that semantic and grammatical class contribute to the organization of the mental lexicon [32]. When comparing object noun and verb, we observed different patterns of activation for the two classes of words. However, the comparison between event noun and verb suggests that when words sharing aspects of knowledge are compared, they may act in similar ways regardless of their grammatical class.

## Materials and Methods

Twelve Italian speakers, students at the University of Trieste, were selected for this study. All subjects were monolingual or had no more than basic skills in one foreign language, as resulting after personal interview. All subjects were healthy volunteers, balanced by gender and age (5 male, 7 female, mean age: 25±2.4 years, min: 21, max: 29), right-handed, and had no visual defects. All the subjects were fully informed on the modality and execution of the scans before signing an informed consent agreement in accordance with the Declaration of Helsinki. Subjects could leave the experiment at any time, although all completed the experimental sessions, and gave written permission to the treatment of personal data. Approval from the Ethics Committee of the University of Trieste was also obtained for this study.

### Materials

Three lists of experimental words were selected: 20 non-argumental nouns (N0) (e.g. *medaglia*-medal), 20 argumental nouns (N1) (e.g. *pianto*-cry), 20 verbs (V) (e.g. *dormire*-to sleep). The complete lists can be found in Appendix S1.

The lists were matched for syllable length (N0: X = 3, SD = 0.86; N1: X = 3.27, SD = 1.08; V: X = 3.27, SD = 0.46) and frequency (N0: X = 26.60, SD = 21.7; N1: X = 24.37, SD = 24.46; V: X = 13.27, SD = 28). They were also matched for familiarity, based on ratings collected from a panel of 18 Italian speakers on a 7-point scale (N0: X = 6.9, SD = 0.06; N1: X = 6.96, SD = 0.09; V: X = 6.96, SD = 0.09).

In addition, two new groups, each of 18 Italian speakers, were interviewed to collect, on a 7-point scale, ratings on concreteness and imageability. Based on the scorings obtained, the lists were also matched for concreteness (N0: X = 5.39, SD = 0.98; N1: X = 5.37, SD = 0.92; V: X = 5.62, SD = 0.93) and imageability (N0: X = 5.57, SD = 0.75; N1: X = 5.38, SD = 1.10; V1: X = 5.87, SD = 1.04).

Finally, an equal number of pseudo-nouns and verbs were selected (e.g. *grincipe* for N0, *darcerazione* for N1, *prigiare* for V) to match with the experimental lists.

### Procedure

The three lists of experimental words were presented in separate MRI sessions. Stimuli were projected in black capital letters on a white screen set in the field of view of the subjects, up-front the MR scanner. Each word was presented for 600 ms; subjects’ attention to the appearance position on screen was aided by using a continuous visual cue (“+”). The interval between subsequent words was semi-randomized to 3, 4, 5 seconds to avoid expectance. The subjects were asked to read the stimuli and to perform a lexical decision task. In order to obtain a measure of this decision, the subjects were instructed to push a comfortable and very sensitive button only when recognizing the word to be part of their vocabulary. Stimuli were presented in three sessions, each including one of the experimental lists and the matching pseudowords. The correctness of the response to stimuli was recorded for further analysis. To minimize head movements during task execution, a number of small cushions were inserted between the subjects’ skull and the head coil after the best and most comfortable position for visual cue was reached.

### Technical Parameters

All images were acquired with a 1.5 Tesla Philips Gyroscan Intera MRI scanner using a multi-channel coil (maximum gradient amplitude: 30 mT/m). The sequence used ramp sampling to minimize distortion and image artifacts. Each f-MRI session consisted in 3750 images (150 volumes, 25 slices each) acquired in about 8 minutes. Parameters were as follow: Fast Field Echo Planar; orientation: axial; TR/TE = 3200/45 ms; flip: 90 degrees; phase encoding: AP; Field of View 210×210 mm; plane resolution: 64×64 pixels; slice thickness: 4 mm; resulting voxel size: 3.28×3.28×4 mm.

### Spatial Pre-processing

Raw images were processed with SPM 5 (The Wellcome Institute, London). We decided to correct for Slice Timing first and then Realign and Unwrap images, because the realignment of raw images showed very little spatial correction (usually less than slice thickness) thus allowing to correct the temporal signal for each voxel without dramatically losing spatial localization [33]–[35]. Timing-corrected and realigned images were spatially normalized to MNI space by using the standard “epi” template. Gaussian smoothing was applied with 8 mm FWHM.

### Statistical Analysis

Pre-processed images were analyzed on an event-related protocol. Pseudowords were taken as baseline. Realignment correction parameters were used as multiple regressors. A high pass temporal filter (cut-off: 128 seconds) was applied to the time series. In1st level analysis, the time series were convolved with the canonical hemodynamic response function (HRF); as the real shape of the HRF may differ from the canonically defined function, the time and dispersion partial derivatives of the HRF were also modeled, in order to identify variations in time occurrence and width of the response to the submitted stimuli, thus increasing detection power [36]–[38]. A t-contrast was defined for every condition and basis (i.e. three contrasts for each condition). The t-contrast images were used to perform F-tests in 2nd level analyses, including global effect (masked by the global effect of pseudowords; threshold p<0.005 uncorrected, minimum extent k = 10 voxels), separate classes effect (masked by the corresponding pseudoword effect; threshold p<0.005 uncorrected, minimum extent k = 10 voxels), and cross comparisons (mutually masked; threshold p<0.05 False Discovery Rate, minimum extent k = 10 voxels). All the resulting areas were checked for positive value of the HRF component. Brodmann areas and brain structures were initially identified with MNI Space utility (MSU, Sergey Pakhomov, www.ihb.spb.ru). The portion of space surrounding each local maximum was then explored with T2T-Muenster Converter (Olaf Steinstrater, www.uni-muenster.de) to ensure proper identification of Brodmann areas in large clusters with more local maxima and clusters in a gap between two contiguous areas.

## Supporting Information

Information S1 1. Global effect 2. Words masked by pseudowords, for each class of words (N0, N1, V) 3. Cross comparisons among classes of words (N0 vs N1, N0 vs V, N1 vs V, and vice-versa) 4. Coupled cross comparisons among classes of words (N0N1 vs V, N0 vs N1V, and vice-versa) 5. Pseudowords masked by words, for each class of words (N0, N1, V).
(DOC)

Appendix S1 **Words and pseudowords as presented in the three experimental sessions (English translation provided for meaningful words).**
(DOC)
