# Supplementary material for: Argument Structure and Morphological Factors in Noun and Verb Processing: An fMRI Study
Source: PLoS One. 2012 Sep 18;7(9):e45091. doi: 10.1371/journal.pone.0045091 (PMC3445573; doi:10.1371/journal.pone.0045091)
Supplement: Information S1 — 1. Global effect 2. Words masked by pseudowords, for each class of words (N0, N1, V) 3. Cross comparisons among classes of words (N0 vs N1, N0 vs V, N1 vs V, and vice-versa) 4. Coupled cross comparisons among classes of words (N0N1 vs V, N0 vs N1V, and vice-versa) 5. Pseudowords masked by words, for each class of words (N0, N1, V). (DOC) [file pone.0045091.s001.doc]

**Supplementary materials**

**Contents:**

1. Global effect

2. Words masked by pseudowords, for each class of words (N0, N1, V)

3. Cross comparisons among classes of words (N0 vs N1, N0 vs V, N1 vs V, and vice-versa)

4. Coupled cross comparisons among classes of words (N0N1 vs V, N0 vs N1V, and vice-versa)

5. Pseudowords masked by words, for each class of words (N0, N1, V)

**1. Global effect:** N0,N1,V words , masked by N0,N1,V pseudowords (p<0.005 uncorrected)


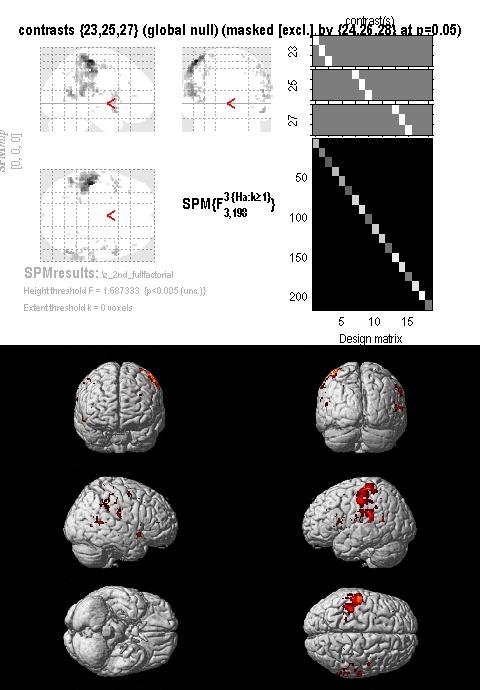

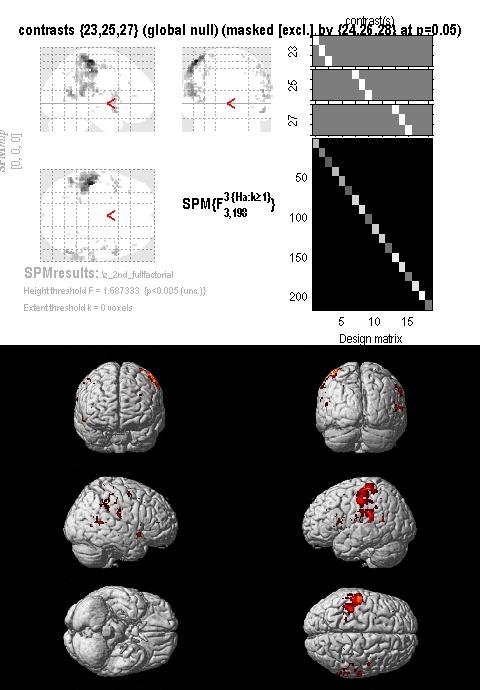


| GLOBAL | Label | Area | x, y, z | k | Z |
| --- | --- | --- | --- | --- | --- |
| Left Hemisphere |  | | | | |
| Frontal lobe | Inferior frontal gyrus and precentral gyrus | L44 | -46 12 10 | 15 | 3.33 |
| Temporal lobe | Superior temporal gyrus | L22 | -48 -54 14 | 44 | 4.38 |
| Parietal lobe | Inferior parietal lobule | L40 | -54 -36 12 | 258 | 4.40 |
|  | Postcentral gyrus | L43 | -56 -14 18 | 10 | 4.14 |
| Right Hemisphere |  | | | | |
| Frontal lobe | Precentral gyrus | R6 | 48 -2 54 | 14 | 3.39 |
| Temporal lobe | Superior temporal gyrus | R38/22 | 52 14 -12 | 27 | 3.82 |
|  | Superior temporal gyrus | R22 | 56 -56 12 | 48 | 3.76 |
| Parietal lobe | Inferior parietal lobule | R40 | 62 -44 42 | 45 | 3.52 |
|  | Postcentral gyrus | R43 | 66 -20 22 | 33 | 3.61 |

**2. Words masked by pseudowords (p<0.005, uncorrected)**

2.1. Object noun masked by object pseudonoun


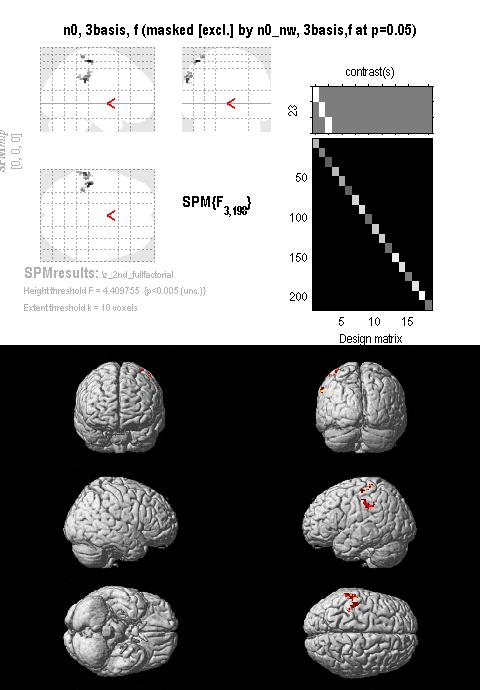

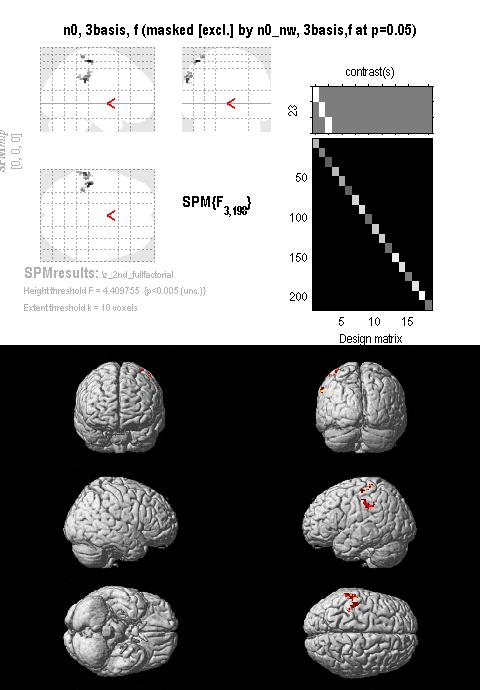


| N0w vs N0nw | Label | Area | x, y, z | k | Z |
| --- | --- | --- | --- | --- | --- |
| Left Hemisphere |  | | | | |
| Parietal lobe | Inferior parietal lobule and postcentral gyrus | L40 | -60 -32 32 | 49 | 3.10 |

2.2. Event noun masked by event pseudonoun


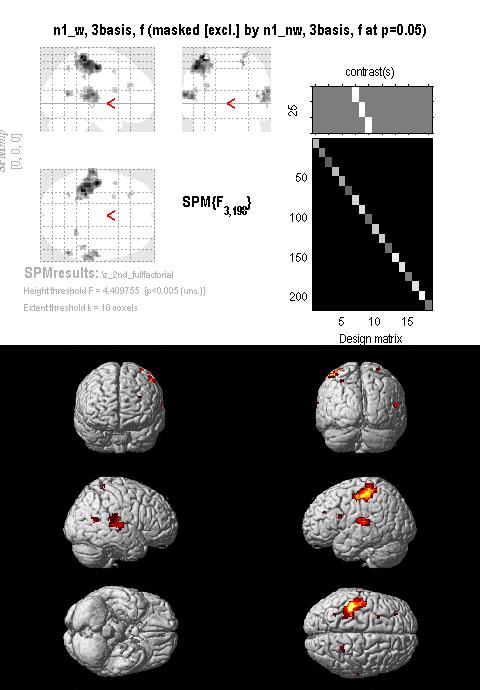

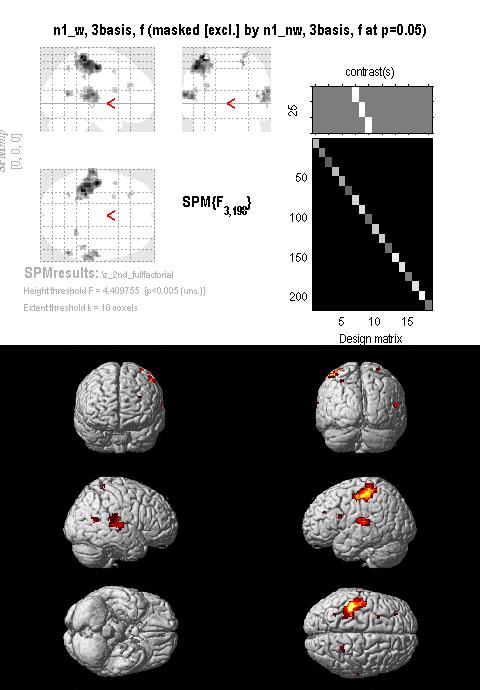


| N1w vs N1nw | Label | Area | x, y, z | k | Z |
| --- | --- | --- | --- | --- | --- |
| Left Hemisphere |  | | | | |
| Frontal lobe | Middle frontal gyrus | L46/10 | -28 32 22 | 34 | 3.00 |
|  | Inferior frontal gyrus | L45 | -44 14 16 | 19 | 3.08 |
|  | Middle frontal gyrus | L8/6 | -26 10 48 | 11 | 2.94 |
| Temporal lobe | Sup. and transv. temp. gy. and postcentral gy. | L40/41/42 | -62 -20 12 | 215 | 4.13 |
| Right Hemisphere |  | | | | |
| Temporal lobe | Superior temporal gyrus | R41/29 | 46 -34 10 | 19 | 3.49 |
|  | Superior and middle temporal gyrus | R39 | 56 -58 14 | 39 | 3.85 |
|  | Superior temporal gy. and postcentral gyrus | R22/40/41/42 | 60 -28 16 | 231 | 3.78 |

2.3. Verb masked by pseudoverb


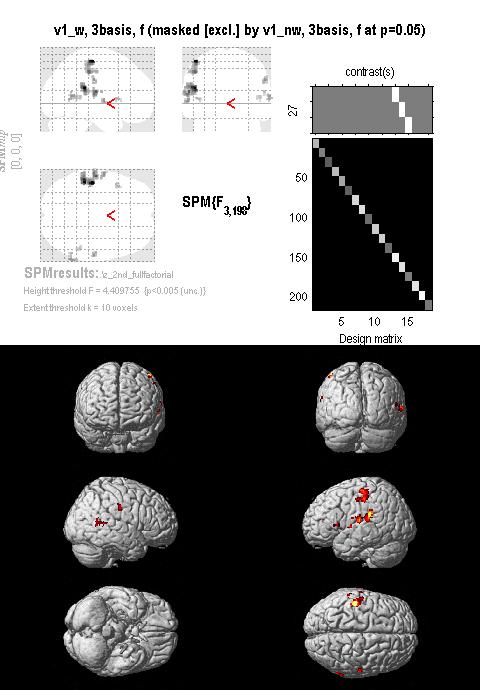

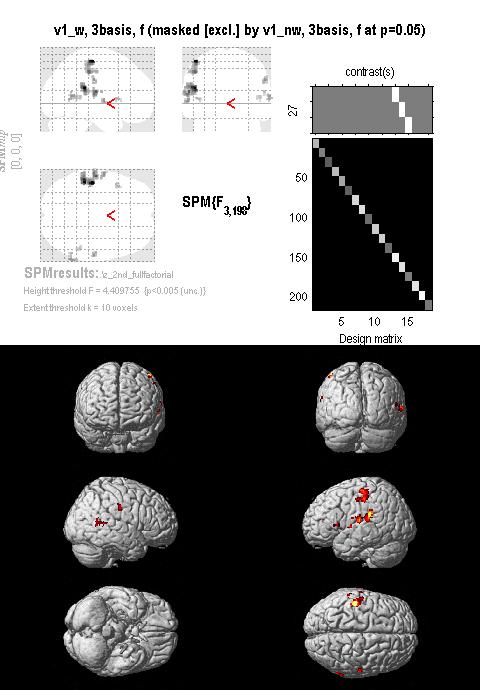


| Vw vs Vnw | Label | Area | x, y, z | k | Z |
| --- | --- | --- | --- | --- | --- |
| Left Hemisphere |  | | | | |
| Frontal lobe | Insula/precentral gyrus/inferior frontal gyrus | L13/44/45 | -42 18 6 | 17 | 2.97 |
|  | Anterior cingulate and middle frontal gyrus | L10/32 | -18 44 12 | 16 | 3.16 |
| Temporal lobe | Superior temporal gyrus | L22 | -58 -4 4 | 33 | 3.24 |
| Parietal lobe | Inferior parietal lobule | L40 | -48 -32 22 | 304 | 4.16 |
|  | Postcentral gyrus | L43 | -56 -6 16 | 10 | 2.99 |
| Right Hemisphere |  | | | | |
| Temporal lobe | Superior temporal gyrus | R22 | 66 -52 10 | 43 | 3.21 |
|  | Superior temporal gyrus | R22 | 50 -40 10 | 23 | 2.86 |
|  | Middle temporal gyrus | R37 | 40 -54 6 | 15 | 3.28 |

In the following figure, the results for words masked by pseudowords are superimposed on the same background. Color codes are: red: object noun; blue: event noun; green: verb.


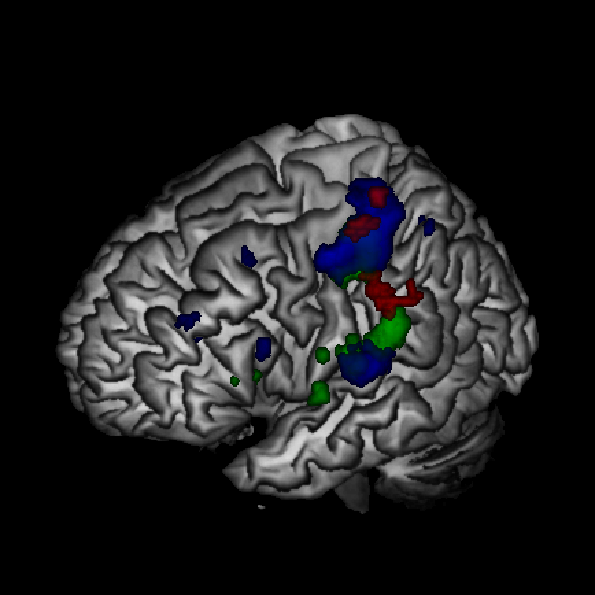

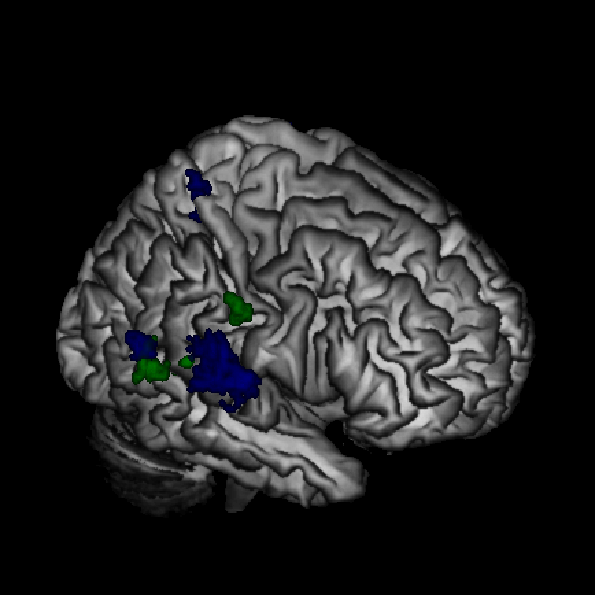


**3. Cross comparisons (3 basis functions, exclusive masking at 0.05, FDR correction, p<0.05)**

3.1. Object noun masked by Event noun


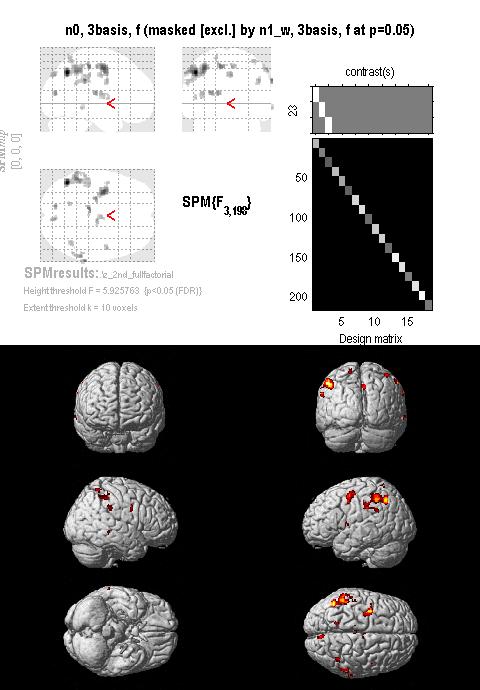

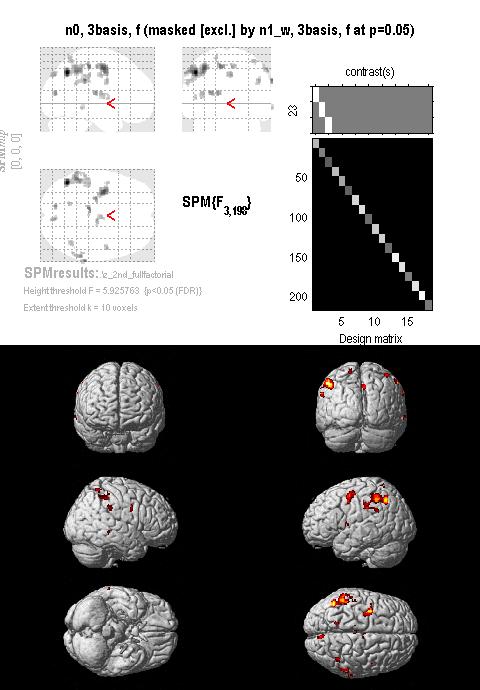


| N0w vs N1w | Label | Area | x, y, z | k | Z |
| --- | --- | --- | --- | --- | --- |
| Left Hemisphere |  | | | | |
| Frontal lobe | Precentral gyrus | L44 | -46 2 8 | 13 | 3.66 |
|  | Insula | L13 | -36 -6 14 | 41 | 3.93 |
|  | Middle frontal gyrus | L6 | -34 -4 52 | 198 | 4.90 |
| Parietal lobe | Inferior parietal lobule | L40 | -46 -60 46 | 469 | 5.64 |
|  | Superior parietal lobule | L7 | -16 -56 66 | 17 | 3.77 |
| Sub-lobar | Ventral lateral nucleus | / | -24 -16 16 | 27 | 4.28 |
| Right Hemisphere |  | | | | |
| Frontal lobe | Inferior frontal gyrus/precentral gyrus | R6/9 | 48 0 34 | 29 | 3.98 |
| Temporal lobe | Middle temporal gyrus | R21 | 68 -36 -6 | 13 | 3.71 |
| Parietal lobe | Inferior parietal lobule | R40 | 68 -32 34 | 24 | 4.59 |
|  | Parietal lobe/precuneus | R7 | 6 -78 42 | 41 | 4.10 |
| Inter-hemispheric | Cingulate gyrus | R24 | 4 -6 42 | 29 | 3.86 |

3.2. Event noun masked by Object noun


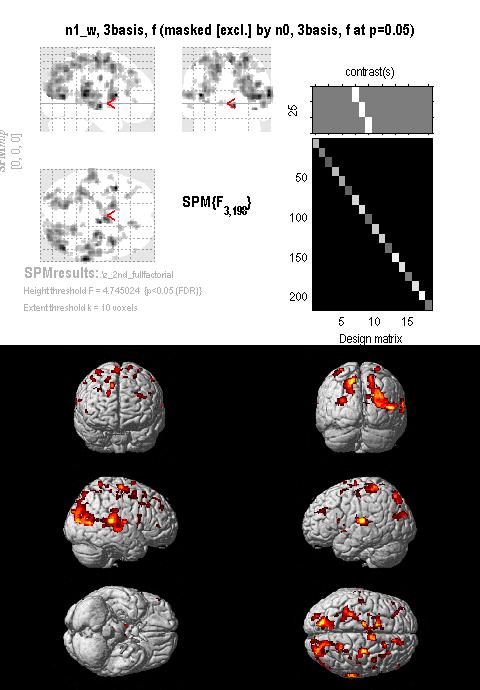

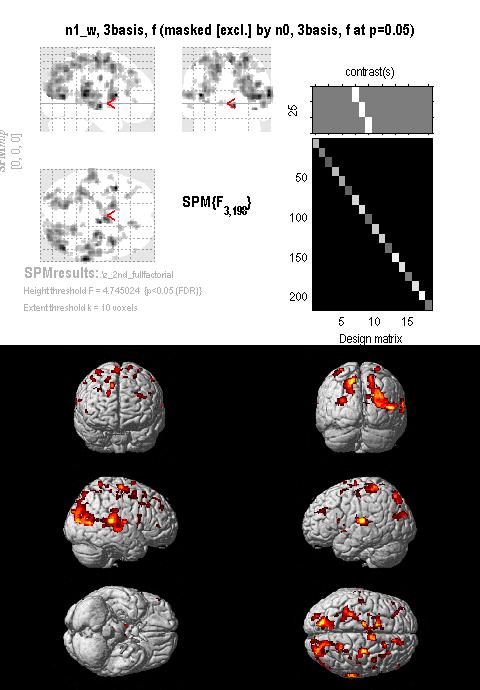


| N1w vs N0w | Label | Area | x, y, z | k | Z |
| --- | --- | --- | --- | --- | --- |
| Left Hemisphere |  | | | | |
| Frontal lobe | Insula/precentral and inferior frontal gyrus | L13/44 | -38 14 12 | 83 | 6.14 |
|  | Middle frontal gyrus | L10/9/46 | -28 30 14 | 152 | 4.12 |
|  | Insula | L13 | -32 -36 18 | 76 | 4.91 |
|  | Middle frontal gyrus | L9/46 | -48 34 34 | 10 | 3.13 |
|  | Middle and superior frontal gyrus | L6 | -30 -4 68 | 12 | 3.15 |
| Temporal lobe | Superior temporal gyrus | L22 | -56 6 -4 | 24 | 3.33 |
|  | Superior temporal gyrus | L41 | -34 -32 4 | 37 | 3.87 |
|  | Superior and transverse temporal gyrus | L41/42 | -56 -18 12 | 321 | 4.59 |
| Parietal lobe | Postcentral gyrus | L40 | -32 -38 66 | 426 | 4.68 |
| Sub-lobar | Parahyppocampal gyrus | L27/30 | -10 -38 0 | 16 | 3.46 |
| Right Hemisphere |  | | | | |
| Frontal lobe | Insula/precentral gyrus | R13 | 40 10 10 | 405 | 5.34 |
|  | Inferior frontal gyrus | R44/45/46 | 54 42 2 | 14 | 3.44 |
|  | Inferior frontal gyrus | R45 | 64 14 22 | 13 | 3.21 |
|  | Superior frontal gyrus | R8/6 | 16 20 56 | 76 | 3.86 |
|  | Precentral/middle frontal gyrus | R6 | 28 -16 66 | 263 | 5.33 |
| Temporal lobe | Superior temporal gyrus/insula | R22/13 | 44 -14 -8 | 28 | 4.74 |
|  | Superior temporal/postcentral gyrus | R22/42 | 64 -32 12 | 681 | 6.13 |
|  | Middle temporal gy./precuneus/sup. pariet. | R7/39 | 48 -66 10 | 1827 | 5.72 |
|  | Superior temporal gyrus/inf. Parietal lobule | R29/41 | 48 -30 28 | 267 | 5.00 |
| Parietal lobe | Postcentral gyrus | R40 | 40 -34 60 | 15 | 3.38 |
| Sub-lobar | Subthalamic nucleus | / | 10 -10 -4 | 41 | 5.72 |

3.3. Object noun masked by Verb


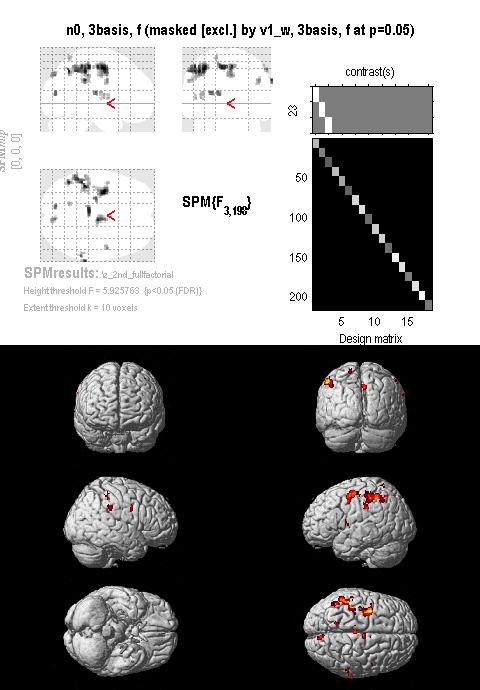

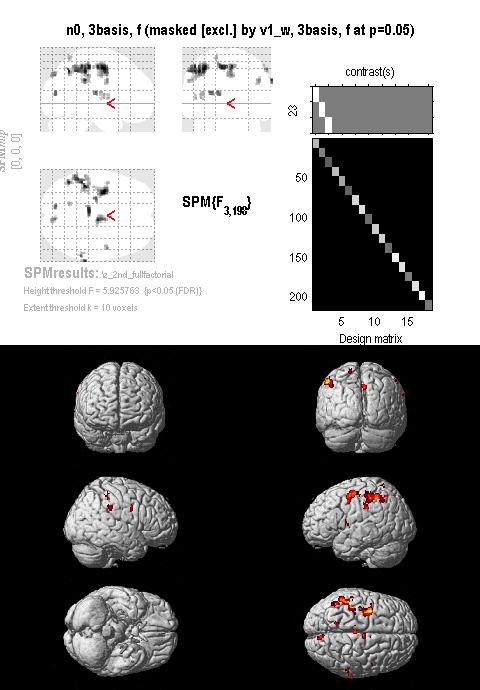


| N0w vs Vw | Label | Area | x, y, z | k | Z |
| --- | --- | --- | --- | --- | --- |
| Left Hemisphere |  | | | | |
| Frontal lobe | Insula | L13 | -36 -8 16 | 31 | 3.77 |
|  | Precentral/inferior frontal gyrus | L44 | -46 2 8 | 15 | 3.66 |
|  | Middle frontal gyrus | L6 | -34 -4 52 | 258 | 4.90 |
| Parietal lobe | Inferior parietal lobule | L40 | -42 -32 48 | 381 | 4.73 |
|  | Superior parietal lobule | L7 | -16 -56 66 | 16 | 3.77 |
| Right Hemisphere |  | | | | |
| Frontal lobe | Precentral/inferior frontal gyrus | L9/6 | 48 0 34 | 30 | 3.98 |
|  | Medial frontal gyrus/cingulate gyrus | R6/24 | 8 -12 54 | 158 | 4.61 |
| Parietal lobe | Inferior parietal lobule | R40 | 68 -32 34 | 27 | 4.59 |
|  | Parietal lobe/precuneus | R7 | 6 -78 42 | 48 | 4.10 |

3.4. Verb masked by Object noun


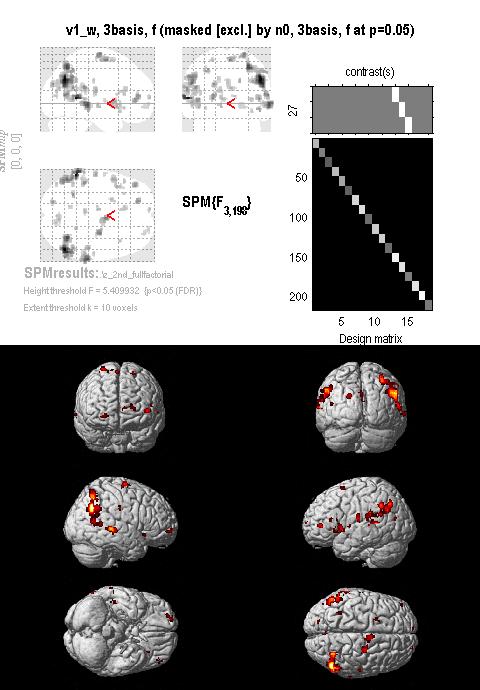

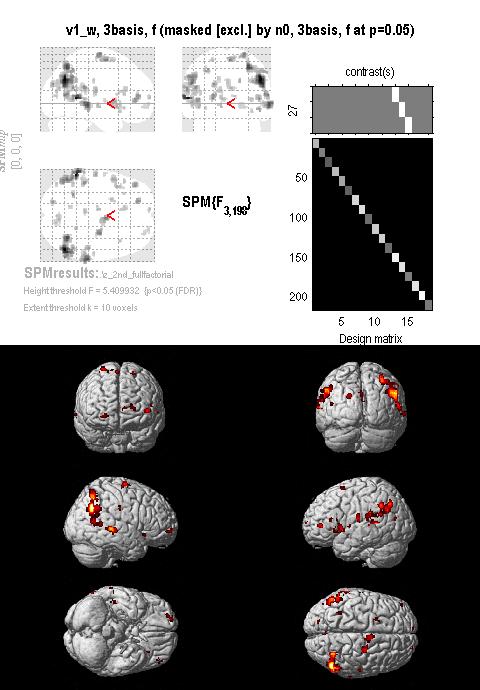


| Vw vs N0w | Label | Area | x, y, z | k | Z |
| --- | --- | --- | --- | --- | --- |
| Left Hemisphere |  | | | | |
| Frontal lobe | Inferior frontal gyrus | L47 | -32 26 -6 | 11 | 3.45 |
|  | Insula/inferior frontal gyrus | L47/13/45 | -52 10 2 | 115 | 3.99 |
|  | Inferior frontal gyrus | L10/46 | -42 42 6 | 51 | 4.25 |
|  | Medial frontal gyrus/anterior cingulate | L10/32 | -20 48 8 | 54 | 4.41 |
|  | Medial frontal gyrus | L10 | -8 66 8 | 17 | 3.71 |
|  | Insula | L13 | -38 16 14 | 13 | 3.47 |
|  | Inferior frontal gyrus | L46 | -48 26 14 | 14 | 3.58 |
|  | Superior frontal gyrus | L6 | -22 8 64 | 23 | 3.68 |
| Temporal lobe | Superior temporal gyrus | L22/6 | -60 -2 6 | 10 | 3.24 |
| Parietal lobe | Inferior parietal lobule/supramarginal gyrus | L40/39 | -58 -46 8 | 385 | 5.78 |
|  | Inferior parietal lobule/supramarginal gyrus | L40 | -46 -50 22 | 17 | 3.70 |
|  | Inferior parietal lobule/insula | L40/13 | -46 -34 24 | 89 | 4.34 |
|  | Postcentral gyrus | L41/42/43 | -58 -16 18 | 59 | 3.99 |
|  | Cuneus/precuneus | L31/7 | -18 -80 28 | 48 | 4.25 |
| Inter-hemispheric | Anterior cingulate | L24 | -10 28 20 | 50 | 3.63 |
| Sub-lobar | Subthalamic nucleus | / | -8 -10 -4 | 13 | 3.72 |
| Right Hemisphere |  | | | | |
| Frontal lobe | Inferior frontal gyrus | R47 | 38 20 -10 | 14 | 3.51 |
|  | Superior frontal gyrus | R10 | 28 56 -4 | 40 | 3.36 |
|  | Medial/superior frontal gyrus | R6 | 4 0 64 | 89 | 4.55 |
| Temporal lobe | Middle temporal gyrus | R21 | 48 -26 -8 | 24 | 4.12 |
|  | Middle temporal gyrus | R21/22 | 64 -28 -2 | 169 | 5.07 |
|  | Superior temporal gyrus | R39 | 48 -50 6 | 44 | 4.19 |
| Parietal lobe | Inferior parietal lobuel/angular gyrus | R39/40 | 52 -62 36 | 726 | 5.97 |
|  | Precuneus | R31 | 20 -46 32 | 12 | 3.83 |

3.5. Event noun masked by Verb


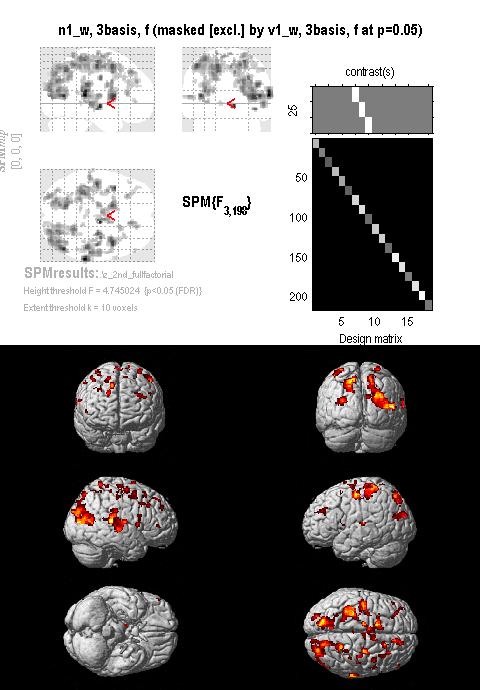

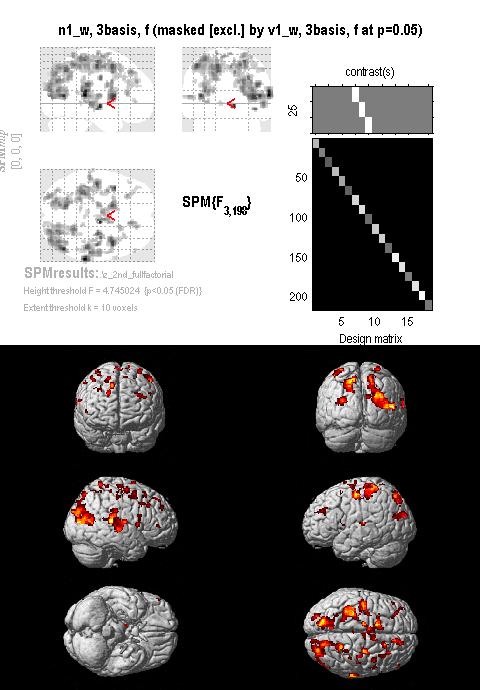


| N1w vs Vw | Label | Area | x, y, z | k | Z |
| --- | --- | --- | --- | --- | --- |
| Left Hemisphere |  | | | |  |
| Frontal lobe | Insula/precentral gyrus | L13/44 | -38 12 10 | 71 | 5.30 |
|  | Middle frontal gyrus | L9/10/46 | -28 30 14 | 148 | 3.48 |
|  | Insula | L13 | -32 -36 18 | 74 | 4.91 |
|  | Precentral gyrus/inferior frontal gyrus | L6/9 | -34 2 28 | 47 | 4.13 |
|  | Middle frontal gyrus | L9/46 | -48 34 34 | 10 | 3.13 |
| Temporal lobe | Superior temporal gyrus | L41 | -34 -32 4 | 35 | 3.87 |
|  | Superior/transverse temporal gyrus | L41/42 | -56 -24 10 | 47 | 4.29 |
| Parietal lobe | Precuneus/superior parietal lobule | L7 | -12 -62 48 | 706 | 4.97 |
| Sub-lobar | Parahyppocampal gyrus | L27/30 | -10 -38 0 | 16 | 3.46 |
| Right Hemisphere |  | | | |  |
| Frontal lobe | Middle and inferior frontal gyrus/insula | R13/44 | 40 10 10 | 448 | 5.34 |
|  | Inferior frontal gyrus | R44/46 | 54 42 2 | 14 | 3.44 |
|  | Inferior frontal gyrus | R45 | 64 14 22 | 13 | 3.21 |
|  | Medial frontal gyrus | R8 | 12 36 48 | 134 | 4.47 |
|  | Middle frontal/precentral gyrus | R6 | 26 -12 62 | 171 | 5.01 |
| Temporal lobe | Insula/superior temporal gyrus | R13/22 | 44 -14 -8 | 31 | 4.74 |
|  | Superior temporal gyrus | R42 | 64 -32 12 | 497 | 6.13 |
| Parietal lobe | Superior parietal gy/cuneus/middle temp.gy. | R7/39 | 50 -64 10 | 1637 | 5.54 |
|  | Postcentral gyrus | R40 | 40 -34 60 | 15 | 3.38 |
| Sub-lobar | Subthalamic nucleus/red nucleus | / | 10 -10 -4 | 25 | 5.72 |

3.6. Verb masked by Event noun


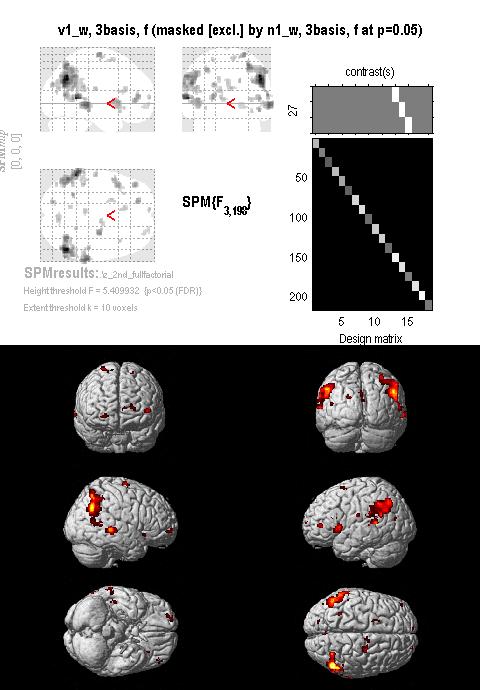

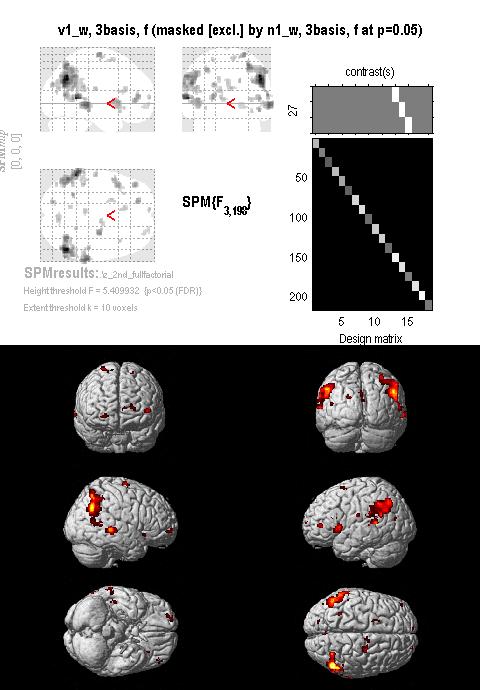


| Vw vs N1w | Label | Area | x, y, z | k | Z |
| --- | --- | --- | --- | --- | --- |
| Left Hemisphere |  | | | | |
| Frontal lobe | Inferior frontal gyrus | L47 | -32 26 -6 | 11 | 3.45 |
|  | Insula/inferior frontal gyrus | L47/13/45 | -42 18 2 | 177 | 4.29 |
|  | Inferior frontal gyrus | L10/46 | -42 42 6 | 51 | 4.25 |
|  | Medial frontal gyrus/anterior cingulate | L10/32 | -20 48 8 | 54 | 4.41 |
|  | Medial frontal gyrus | L10 | -8 66 8 | 17 | 3.71 |
|  | Superior frontal gyrus | L6 | -22 8 64 | 21 | 3.68 |
| Temporal lobe | Superior temporal gyrus | L22/42 | -58 -46 8 | 93 | 5.78 |
| Parietal lobe | Inferior parietal lobule/supramarginal gyrus | L40 | -52 -58 30 | 822 | 5.05 |
|  | Postcentral gyrus | L43 | -58 -16 20 | 21 | 3.91 |
|  | Cuneus/precuneus | L31/7 | -18 -80 28 | 35 | 4.25 |
|  | Superior parietal lobule | L7 | -28 -50 50 | 33 | 4.25 |
| Inter-hemispheric | Anterior cingulate | L24 | -10 28 20 | 50 | 3.63 |
| Right Hemisphere |  | | | | |
| Frontal lobe | Inferior frontal gyrus | R47 | 38 20 -10 | 14 | 3.51 |
|  | Superior frontal gyrus | R10 | 28 56 -4 | 40 | 3.36 |
|  | Medial/superior frontal gyrus | R6 | 10 -6 66 | 19 | 3.60 |
| Temporal lobe | Middle temporal gyrus | R21 | 48 -26 -10 | 24 | 4.15 |
|  | Middle temporal gyrus | R21/22 | 64 -28 -2 | 204 | 5.07 |
|  | Superior temporal gyrus | R39 | 50 -50 8 | 59 | 4.24 |
| Parietal lobe | Inferior parietal lobuel/angular gyrus | R40 | 52 -60 36 | 888 | 6.19 |
|  | Precuneus | R31 | 6 -72 22 | 78 | 3.81 |

**4. Coupled cross comparisons (3 basis functions, exclusive masking at 0.05, FDR correction, p<0.05)**

4.1. Object and Event noun masked by Verb


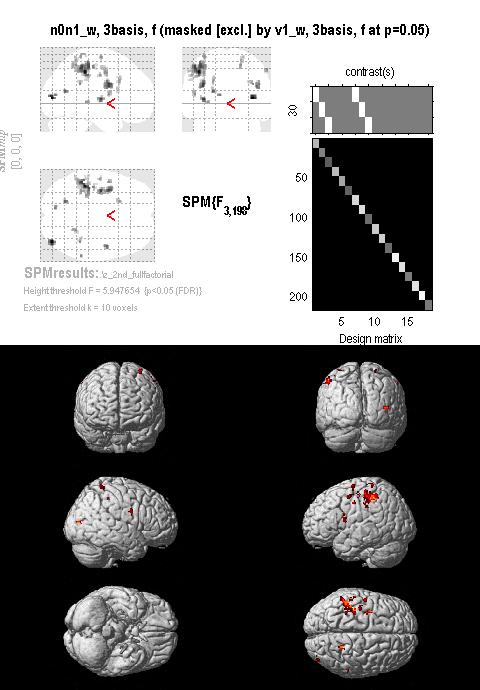

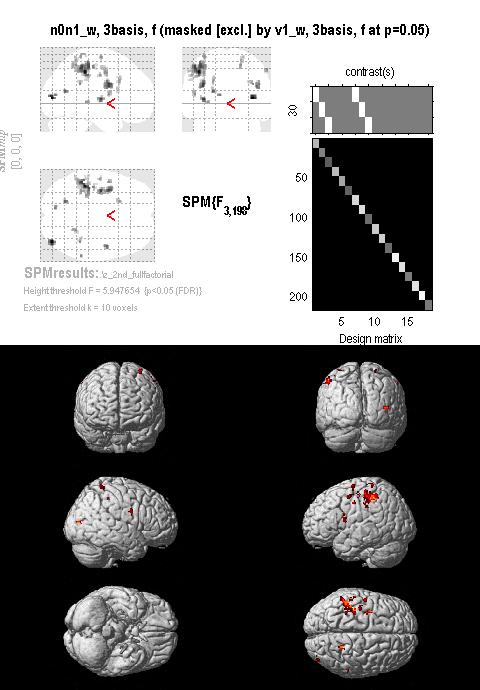


| N0N1w vs Vw | Label | Area | x, y, z | k | Z |
| --- | --- | --- | --- | --- | --- |
| Left Hemisphere |  | | | | |
| Frontal lobe | Insula/claustrum | 13 | -34 -8 6 | 37 | 4.59 |
|  | Precentral/inferior frontal gyrus | L44 | -48 4 14 | 20 | 3.98 |
|  | Precentral/inferior frontal gyrus | L9/6 | -36 4 26 | 80 | 4.59 |
|  | Middle frontal gyrus | L6 | -34 -2 46 | 51 | 3.99 |
| Parietal lobe | Inferior parietal lobule | L40 | -42 -32 46 | 303 | 4.92 |
| Inter-hemispheric | Anterior cingulate cortex | L32 | -20 18 32 | 21 | 3.87 |
| Right Hemisphere |  | | | | |
| Frontal lobe | Precentral/inferior frontal gyrus | L9/6 | 46 0 26 | 38 | 4.32 |
| Temporal lobe | Middle temporal gyrus | R39 | 40 -82 8 | 41 | 5.07 |

4.2. Verb masked by Object and Event nouns


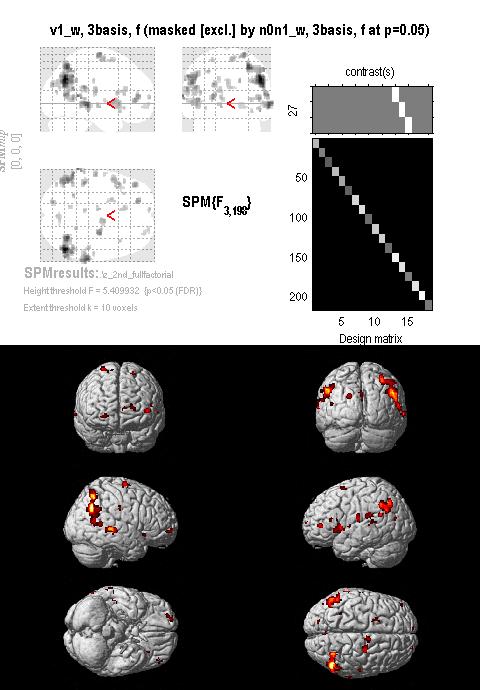

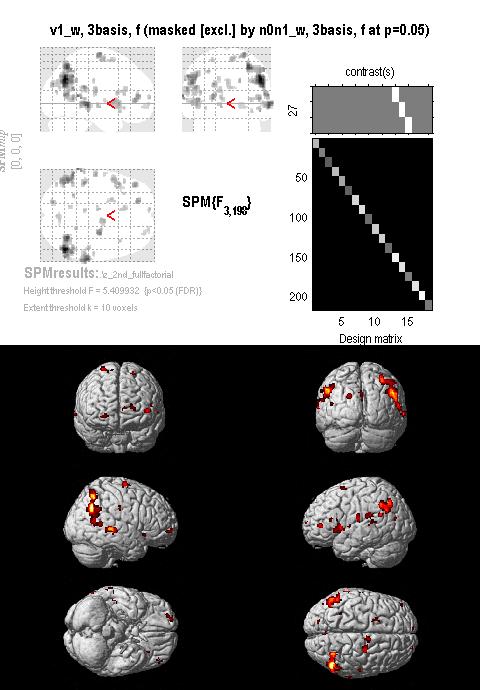


| Vw vs N0N1w | Label | Area | x, y, z | k | Z |
| --- | --- | --- | --- | --- | --- |
| Left Hemisphere |  | | | | |
| Frontal lobe | Inferior frontal gyrus | L47 | -32 26 -6 | 11 | 3.45 |
|  | Inferior frontal gyrus/insula | L47/13 | -44 18 0 | 145 | 4.18 |
|  | Inferior frontal gyrus | L10/46 | -42 42 6 | 51 | 4.25 |
|  | Medial frontal gyrus/anterior cingulate | L10/32 | -20 48 8 | 54 | 4.41 |
|  | Medial frontal gyrus | L10 | -8 66 8 | 17 | 3.71 |
|  | Insula | L13 | -38 16 16 | 18 | 3.84 |
|  | Inferior frontal gyrus | L46 | -48 26 14 | 14 | 3.58 |
|  | Medial frontal gyrus | L6 | -22 8 64 | 31 | 3.68 |
| Temporal lobe | Superior temporal gyrus | L22 | -60 -2 6 | 10 | 3.24 |
|  | Angular gyrus/supramarginal gyrus | L39/40 | -50 -58 30 | 341 | 4.72 |
| Parietal lobe | Superior temporal/inferior parietal lobule | L22/42/40 | -66 -42 10 | 30 | 4.68 |
|  | Postcentral gyrus | L43/42/41 | -58 -16 18 | 61 | 3.99 |
|  | Inferior parietal lobe/insula | L40/13 | -46 -34 24 | 68 | 4.34 |
|  | Supramarginal gyrus/inferior parietal lobule | L40 | -48 -52 22 | 30 | 3.92 |
|  | Precuneus | L31 | -12 -56 38 | 13 | 3.47 |
| Sub-lobar | Subthalamic nucleus | / | -8 -10 -4 | 14 | 3.72 |
|  | Pulvinar | / | -6 -32 6 | 13 | 3.64 |
| Right Hemisphere |  | | | | |
| Frontal lobe | Inferior frontal gyrus | R47 | 38 20 -10 | 14 | 3.51 |
|  | Superior frontal gyrus | R10 | 28 56 -4 | 40 | 3.36 |
|  | Medial frontal gyrus | R6 | 10 -4 66 | 46 | 3.89 |
| Temporal lobe | Middle/superior temporal gyrus | R21 | 50 -28 -10 | 18 | 3.98 |
|  | Middle/superior temporal gyrus | R21/22 | 64 -28 -2 | 185 | 5.07 |
|  | Superior temporal gyrus | R39/7 | 52 -62 36 | 744 | 5.97 |
| Parietal lobe | Precuneus | R31 | 2 -74 30 | 59 | 3.58 |

4.3. Object noun masked by Event noun and Verb


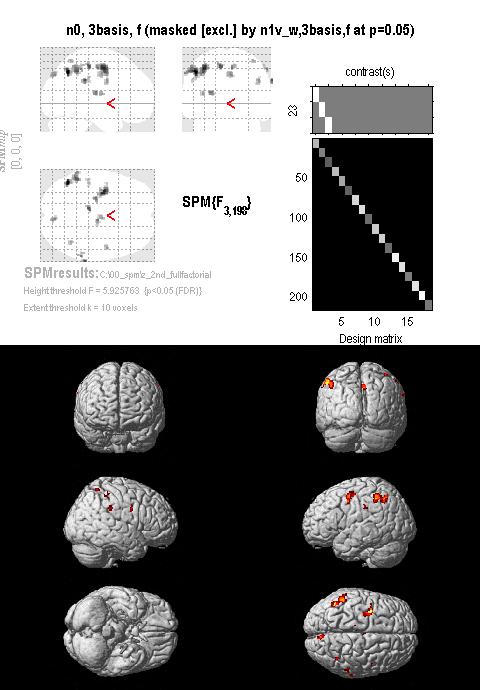

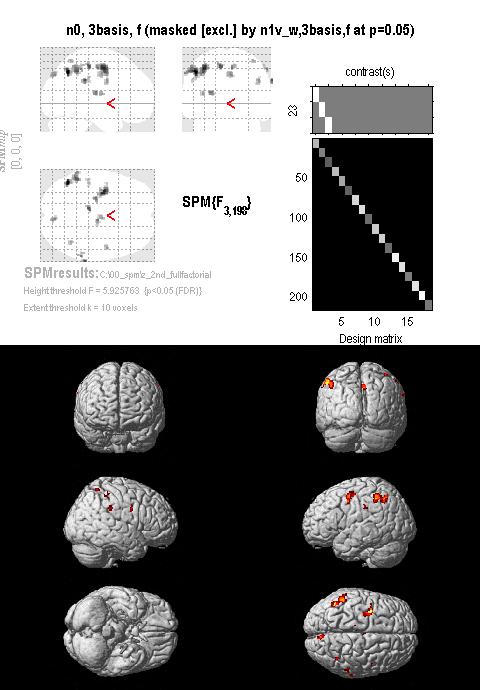


| N0w vs N1Vw | Label | Area | x, y, z | k | Z |
| --- | --- | --- | --- | --- | --- |
| Left Hemisphere |  | | | | |
| Frontal lobe | Insula | L13 | -36 -6 14 | 17 | 3.93 |
|  | Middle Frontal Gyrus | L6 | -34 -4 52 | 163 | 4.90 |
|  | Medial Frontal Gyrus | L6 | -10 -26 54 | 35 | 4.51 |
| Parietal lobe | Inferior Parietal Lobule/Postcentral gyrus | L40 | -46 -26 38 | 24 | 3.73 |
|  | Inferior Parietal Lobule | L40 | -48 -58 48 | 183 | 5.19 |
| Sub-lobar | Ventral Lateral Nucleus | / | -24 -16 16 | 26 | 4.28 |
| Right Hemisphere |  | | | | |
| Frontal lobe | Inferior Frontal Gyrus | R9 | 48 0 34 | 25 | 3.98 |
|  | Cingulate Gyrus | R24 | 4 -6 44 | 40 | 3.96 |
|  | Medial Frontal Gyrus | R6 | 8 -12 52 | 23 | 4.28 |
| Parietal lobe | Inferior Parietal Lobule | R40 | 68 -32 34 | 19 | 4.59 |
|  | Precuneus | R7 | 6 -78 42 | 43 | 4.10 |
|  | Inferior Parietal Lobule/Postcentral gyrus | R40 | 60 -38 50 | 20 | 4.18 |

4.4. Event noun and Verb masked by Object noun


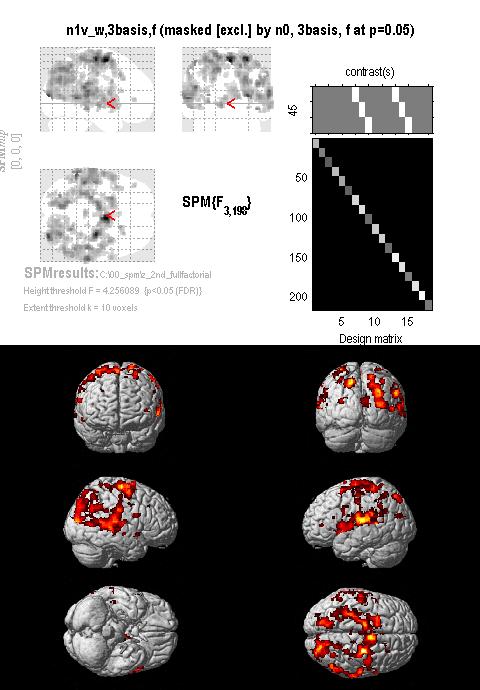

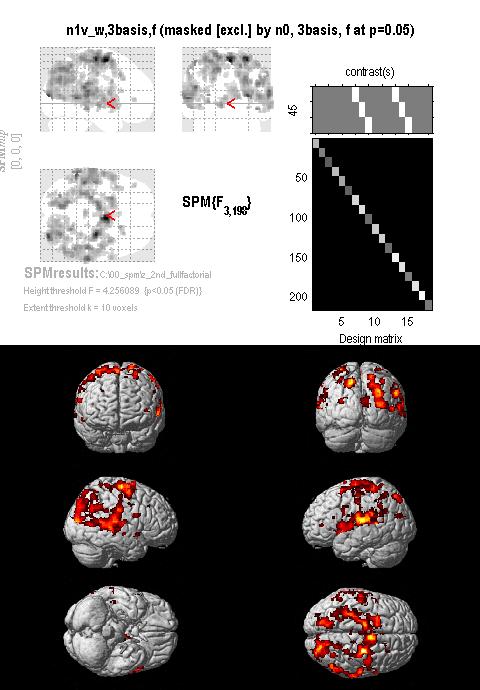


| N1Vw vs N0w | Label | Area | x, y, z | k | Z |
| --- | --- | --- | --- | --- | --- |
| Left Hemisphere |  | | | | |
| Frontal lobe | Insula/Precentral Gyrus | L13/44 | -38 14 10 | 22 | 3.48 |
|  | Inferiior Frontal Gyrus | L9/44 | -46 8 30 | 67 | 2.95 |
|  | Cingulate Gyrus | L32 | -2 20 40 | 35 | 2.91 |
|  | Inferior Frontal Gyrus | L45 | -54 16 4 | 5 | 3.09 |
|  | Inferior Frontal Gyrus | L46 | -42 42 6 | 21 | 3.55 |
|  | Inferior Frontal Gyrus | L46 | -38 24 18 | 22 | 3.26 |
| Temporal lobe | Sup. Tem. Gy./Transverse Tem. Gy. Precentr. Gy./ Postcentr Gy./Insula/Inf.Par.Lobule | L22/40/42/43/  13/45 | -58 -46 8 | 2212 | 5.44 |
|  | Transverse/Superior temporal Gyrus | L41 | -26 -26 2 | 59 | 4.25 |
| Parietal lobe | Superior Parietal Lobule/Precuneus | L7 | -12 -64 50 | 650 | 4.89 |
|  | Inferior/Superior Parietal Lobule | L7/40 | -34 -50 48 | 161 | 3.84 |
| Sub-lobar | Ventral lat. Nucl / Medial globus pallidus | / | -6 -10 -2 | 94 | 4.41 |
| RightHemisphere |  | | | | |
| Frontal lobe | Insula | R13 | 40 4 16 | 171 | 4.16 |
|  | Inferior Frontal Gyrus | R44/45 | 62 6 22 | 59 | 3.70 |
|  | Cingulate Gyrus | R32 | 18 22 42 | 86 | 4.13 |
|  | Inferior Frontal Gyrus | R45 | 62 10 22 | 12 | 3.35 |
| Temporal lobe | Superior Temporal Gyrus | R38 | 58 6 -12 | 41 | 3.14 |
|  | Middle Temporal Gyrus | R22 | 56 -12 -10 | 19 | 3.29 |
|  | Middle/Superior Temp. Gy., Postcentral Gy. | R22/21/42 | 64 -28 18 | 1825 | 5.31 |
|  | Middle Temporal Gyrus | L39 | 48 -64 8 | 110 | 4.30 |
| Parietal lobe | Superior Parietal Lobule/Cuneus | R7 | 32 -86 10 | 1576 | 5.65 |
|  | Supramarginal Gyrus/Inf. Par. Lobule | R40/39 | 54 -62 30 | 332 | 4.43 |
|  | Superior/Inferior Parietal lobule | R7/40 | 34 -60 50 | 82 | 3.86 |
| Sub-lobar | Caudate tail | / | 32 -30 -6 | 13 | 3.64 |

**5. Pseudowords masked by words (p<0.005, uncorrected)**

5.1. Object pseudonoun masked by Object noun


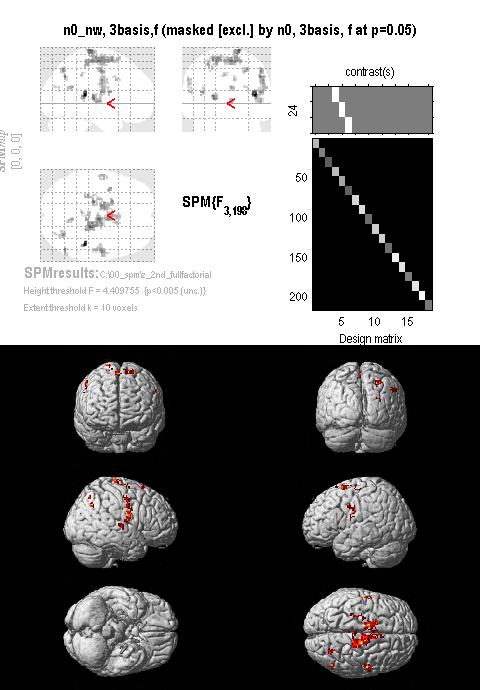

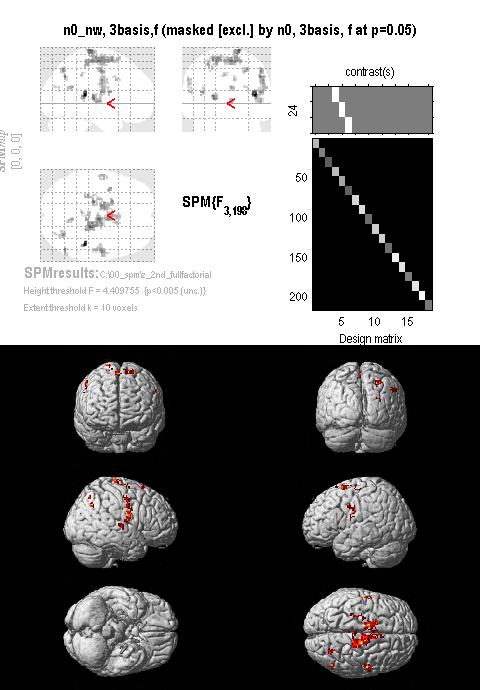


| N0nw vs N0w | Label | Area | x, y, z | k | Z |
| --- | --- | --- | --- | --- | --- |
| Left Hemisphere |  | | | | |
| Frontal lobe | Insula | L13 | -36 4 16 | 12 | 3.35 |
|  | Middle frontal gyrus | L6 | -22 -12 50 | 11 | 3.03 |
|  | Middle/superior frontal gyrus | L6 | -28 -28 58 | 32 | 3.26 |
|  | Cingulate gyrus | L31 | -2 -36 42 | 10 | 3.07 |
| Sub-lobar | Ventral lateral/ Ventr.post. lat. Nucleus | / | -18 -18 6 | 32 | 3.61 |
| Right Hemisphere |  | | | | |
| Frontal lobe | Cingulate gyrus | R32 | 10 16 34 | 17 | 3.01 |
| Temporal lobe | Precentral gy/Superior temporal gyrus | R6,13,22 | 52 -6 48 | 198 | 3.45 |
|  | Superior/transverse temp. Gy/Insula | R41,13,29 | 42 -32 12 | 51 | 4.40 |
| Parietal lobe | Inferior parietal lobule | R40 | 52 -62 38 | 17 | 3.47 |
|  | Superior parietal lobule | R7 | 30 -66 50 | 33 | 3.37 |
|  | Posterior cingulate gyrus | R31,23 | 6 -52 24 | 23 | 3.20 |
| Sub-lobar | Medial dorsal nucleus | / | 6 -10 4 | 21 | 3.25 |

5.2. Event pseudonoun masked by Event noun


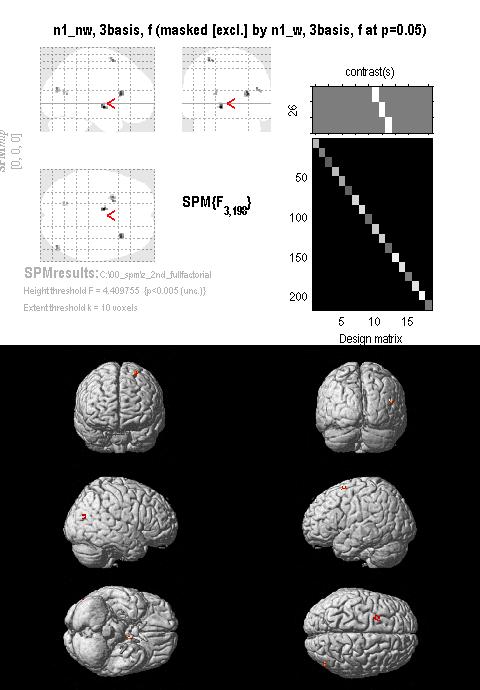

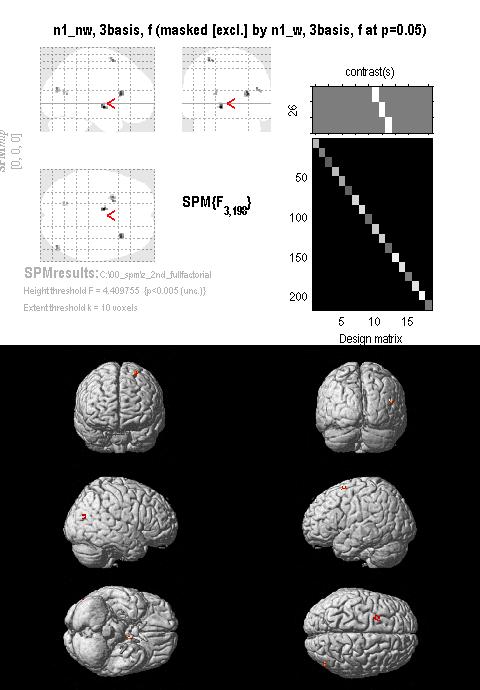


| N1nw vs N1w | Label | Area | x, y, z | k | Z |
| --- | --- | --- | --- | --- | --- |
| Left Hemisphere |  | | | | |
| Frontal lobe | Superior frontal gyrus | L6 | -26 10 64 | 14 | 3.21 |
| Sub-lobar | Medial globus pallidus/hypothalamus | / | -8 -4 -4 | 15 | 3.70 |
| Right Hemisphere |  | | | | |
| Frontal lobe | Insula | R13 | 32 22 14 | 27 | 3.46 |
| Temporal lobe | Middle temporal gyrus | R39 | 46 -74 22 | 11 | 3.12 |

5.3. Pseudoverb masked by Verb


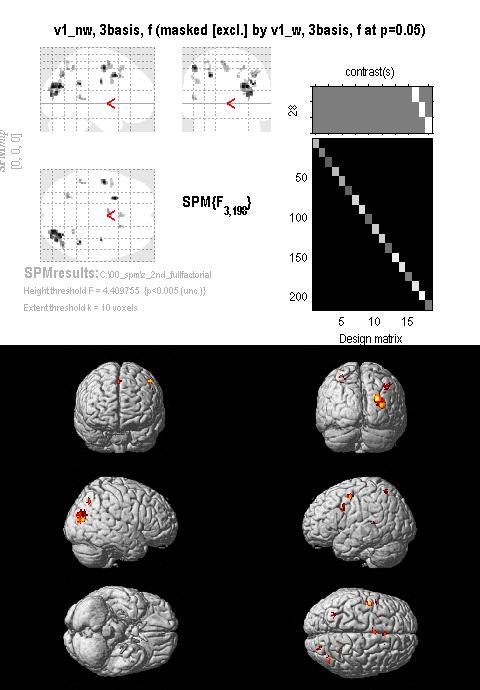

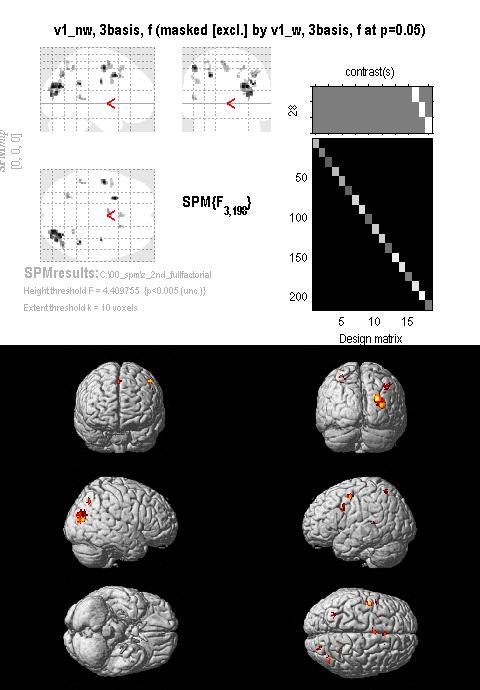


| Vnw vs Vw | Label | Area | x, y, z | K | Z |
| --- | --- | --- | --- | --- | --- |
| Left Hemisphere |  | | | | |
| Frontal lobe | Inferior/middle frontal gyrus | L9 | -44 8 32 | 27 | 2.82 |
|  | Precentral/middle frontal gyrus | L6 | -48 -4 52 | 44 | 3.63 |
|  | Superior/Medial frontal gyrus | L6 | -2 4 58 | 21 | 3.03 |
| Parietal lobe | Superior parietal lobule | L7 | -26 -58 50 | 11 | 3.06 |
|  | Superior parietal lobule | L7 | -28 -60 60 | 11 | 2.87 |
| Right Hemisphere |  | | | | |
| Frontal lobe | Cingulate gyrus | R32 | 16 20 40 | 10 | 3.07 |
| Temporal lobe | Middle temporal gyrus/cuneus | R31 | 30 -72 24 | 260 | 3.70 |
| Parietal lobe | Inferior parietal lobule | R39,40,7 | 44 -66 42 | 32 | 3.25 |
