# Supplementary material for: Argument Structure and Morphological Factors in Noun and Verb Processing: An fMRI Study
Source: PLoS One. 2012 Sep 18;7(9):e45091. doi: 10.1371/journal.pone.0045091 (PMC3445573; doi:10.1371/journal.pone.0045091)
Supplement: Appendix S1 — Words and pseudowords as presented in the three experimental sessions (English translation provided for meaningful words). (DOC) [file pone.0045091.s002.doc]

**Appendix S1**

Words and pseudowords as presented in the three experimental sessions (English translation provided for meaningful words).

*Object nouns*: ligume, musolo, accento (accent), pesca (peach), furina, formica (ant), nuvoda, ferita (gash), terremoto (earthquake), tarruga, fiambo, timbro (stamp), sopeti, lama (blade), cameriere (waiter), davioni, sigore, lati (sides), grincipe, bistone, banda (band), fiocco (bow), vaprina, divisione (division), indise, orizzonte (horizon), frelto , rugola , profumo (scent), scontro (clash), taramo, alfabeto (alphabet), sadile, microfono (microphone), angolo (angle), perolio, risprezzo, medaglia (medal), uceli, insalata (salad).

*Event nouns*: lotta (fight), sbinimento, ruolamento, arresto (arrest), forpata, salconte, brivido (chill), esate, nemilo, duello (duel), furto (theft), abracio, scelta (choice), nuoto (swimming), muscino, inseguimento (chase), ropigliatura, pariga, lucidatura (burnish), passeggiata (walk), zinaro, punizione (punishment), risata (laugh), nistazione, fucisa, sosta (pause), uofo, lavaggio (wash), berimento, liberazione (liberation), romozione, tesunzione, urlo (shout), sparatoria (gunfight), luficazione, pianto (cry), pugnalata (stab), ideramento, darcerazione, pattinaggio.

*Verbs*: piangere (to cry), sorridere (to smile), misedere, nivertire, cenare (to dine), fenzionare, stirare (to iron), predere, difiutare, giurare (to swear), latare, urlare (to shout), gocciolare (to drop), zuotare, prigiare, socitare, nuotare (to swim), brillare (to shine), sartire, miagolare (to meow), disbondere, bussare (to knock), passeggiare (to walk), adevare, gianducere, mottare, ridere (to laugh), ruggire (to rave), sillevare, fischiare (to whistle), bicciare, russare (to snore), ghinare, vicevere, bollire (to boil), ringere, pattinare (to skate), lumpare, sudare (to sweat), suonare (to play).
